# Recent Advances in Polymeric Delivery Vehicles for Controlled and Sustained Drug Release

**DOI:** 10.3390/pharmaceutics16091184

**Published:** 2024-09-07

**Authors:** Hong Lu, Zheng Cai, Ping Hu

**Affiliations:** 1 Department of Burns & Plastic Surgery, Guangzhou Red Cross Hospital, Faculty of Medical Science, Jinan University, Guangzhou 510006, China; luhong0910@foxmail.com; 2 College of Pharmacy, Jinan University, Guangzhou 510006, China; 3 School of Pharmaceutical Sciences, Southern Medical University, Guangzhou 510515, China

## 1. Introduction

In the realm of modern therapeutics, the development of polymeric delivery vehicles has revolutionized drug administration, offering a sophisticated approach to controlled and sustained drug release. Polymeric drug delivery systems provide numerous advantages over traditional delivery methods, including the ability to improve drug stability, enhance bioavailability, and achieve targeted delivery to specific tissues or organs [1,2]. These systems are particularly vital in treating chronic diseases, where maintaining a consistent drug concentration within the therapeutic window is crucial for efficacy and patient compliance.

The concept of controlled drug release involves the gradual release of a drug over time, ensuring prolonged therapeutic effects and reducing the frequency of drug administration. This approach not only enhances patient convenience but also minimizes the risk of side effects associated with peak drug concentrations. Polymeric materials play a pivotal role in this process due to their versatile properties, such as biocompatibility, biodegradability, and tunable mechanical strength. These materials can be engineered to respond to various physiological stimuli, such as pH, temperature, or enzymatic activity, allowing for the precise control of drug release rates [3]. Among the most prominent polymeric systems are hydrogels, microspheres, nanoparticles, and micelles, each offering unique advantages depending on the therapeutic application. Hydrogels, for instance, are highly hydrated networks that can encapsulate a large amount of drug, providing a matrix for sustained release [4]. Microspheres and nanoparticles, on the other hand, offer the possibility of targeted delivery, where the drug is directed to a specific site within the body, thereby enhancing its therapeutic efficacy and reducing systemic exposure [5].

Recent advances in polymer science have led to the development of smart polymers, which can undergo reversible changes in response to environmental stimuli. These materials are particularly promising for creating drug delivery systems that can release their payload in response to specific triggers, such as a change in pH or temperature. This targeted approach not only improves the therapeutic outcomes but also reduces the risk of off-target effects, making these systems highly desirable in the treatment of complex diseases like cancer and neurological disorders [6,7].

Moreover, the integration of polymeric delivery systems with advanced technologies such as 3D printing and nanotechnology has opened up new avenues for personalized medicine. Three-dimensional printing, for example, allows for the fabrication of patient-specific drug delivery devices, tailored to the unique anatomical and physiological characteristics of the individual. This level of customization ensures optimal drug release profiles and improves patient outcomes [8]. Nanotechnology, on the other hand, has enabled the development of polymeric nanoparticles that can cross biological barriers, such as the blood–brain barrier, to deliver drugs to otherwise inaccessible sites. These nanoparticles can be functionalized with targeting ligands that recognize and bind to specific receptors on the surface of diseased cells, ensuring that the drug is delivered precisely where it is needed [9].

The growing body of research in polymeric drug delivery systems reflects the increasing recognition of their potential to address the limitations of traditional drug formulations. For instance, hydrophobic drugs, which are poorly soluble in water, can be encapsulated within polymeric matrices, thereby enhancing their solubility and bioavailability. Similarly, drugs with short half-lives can be formulated into sustained-release systems, reducing the frequency of administration and improving patient adherence.

As we delve into the specific advancements reported in the recent literature, it becomes evident that the field of polymeric drug delivery is on the cusp of significant breakthroughs. The following sections will provide a detailed examination of recent studies that highlight the diverse applications and innovative approaches being developed to enhance controlled and sustained drug release. These studies not only showcase the versatility of polymeric materials but also underscore the ongoing efforts to refine and optimize drug delivery systems for a wide range of therapeutic areas.

## 2. Overview of the Published Articles

The 11 papers in this Special Issue collectively highlight significant advancements in polymeric and nanoparticulate drug delivery systems, showcasing a wide range of innovative approaches aimed at enhancing therapeutic efficacy, stability, and targeted delivery. These studies represent a concerted effort to overcome the challenges associated with conventional drug delivery methods, pushing the boundaries of what is possible in terms of sustained release, targeting specific tissues, and improving the overall bioavailability of therapeutic agents.

Several papers focus on the development of long-acting injectable formulations, which are crucial for ensuring prolonged therapeutic effects and reducing the frequency of drug administration. For example, one study presents a water-free, PLGA-based depot gel designed for the sustained release of the peptide drug ACTY116 (contribution 1). This formulation not only demonstrates improved chemical and conformational stability of the peptide but also exhibits long-acting characteristics that make it an ideal candidate for in vivo sustained peptide delivery. Another study, employing a quality by design (QbD) approach, delves into the degradation mechanisms of peptides in long-acting injections (contribution 2). By conducting forced degradation studies, the researchers identify critical factors that influence peptide stability and implement strategic control measures to enhance the pharmacokinetic profile of the formulation, ensuring prolonged therapeutic efficacy.

In the realm of real-time drug release monitoring, a study utilizing fluorescence resonance energy transfer (FRET) technology makes significant strides (contribution 3). By encapsulating drugs within PLGA microspheres and tagging them with fluorescence markers such as Cy5 and Cy7, the researchers are able to correlate changes in FRET fluorescence with the drug release profile, offering a powerful tool for in vitro drug delivery studies. This method simplifies the analysis of drug release kinetics, providing valuable insights that could lead to better-designed delivery systems.

This Special Issue also emphasizes the development of targeted and responsive drug delivery systems. For instance, the creation of dual-targeted, pH-sensitive hybrid polymer micelles specifically for breast cancer treatment represents a significant advance in targeted therapy (contribution 4). These micelles, constructed from polymers modified with hyaluronic acid and folic acid, not only improve drug stability but also enhance the targeting of cancer cells, ensuring that the drug is released specifically in the tumor microenvironment. Another study introduces a thermo-responsive hydrogel system that encapsulates folate-targeted, curcumin-loaded nanoparticles (contribution 5). This innovative system offers the potential for highly localized drug delivery, especially in post-operative cancer treatment settings, where controlled and sustained release is critical.

Bone regeneration, another vital area of research, is addressed through the use of 3D-printed polycaprolactone (PCL) scaffolds enhanced with nano-topographically guided biomineralized coatings (contribution 6). These scaffolds demonstrate superior protein adsorption, ion release capacities, and the ability to guide the osteogenic differentiation of stem cells, making them a promising solution for bone regeneration therapies. Such advancements highlight the potential of integrating biomaterials with advanced manufacturing techniques to create next-generation therapeutic scaffolds.

Additionally, research exploring the influence of polymer composition on drug release profiles provides essential insights for the design of future delivery systems. One study, in particular, examines how varying the molecular weight and concentration of poly(ethylene) glycol diacrylate (PEGDA) affects the release of a model protein drug from photopolymerized systems (contribution 7). By manipulating these parameters, the researchers are able to modulate the swelling behavior and drug release profiles, thus offering a customizable approach to protein-loaded delivery systems.

This Special Issue also addresses the scalability of advanced drug delivery systems, as demonstrated in a pilot study on solid lipid nanoparticle-based metered-dose inhalers (SLN-MDIs) for lung disease treatment (contribution 8). This research not only demonstrates the successful fabrication of SLN-based MDIs with stable physicochemical properties but also highlights their potential for industrialization and clinical application, marking a significant step towards bringing these innovations from the lab to the marketplace.

Furthermore, the versatility and potential of biopolymer coatings in drug delivery are reviewed in a comprehensive analysis of cyclodextrin/citric acid (CD/CTR) biopolymers (contribution 9). This review underscores the environmentally friendly synthesis, biocompatibility, and effective controlled release mechanisms of CD/CTR-based systems, which hold promise for various drug delivery applications. Similarly, the application of the Box–Behnken design in optimizing tadalafil-loaded niosomal films for oral delivery represents a significant improvement in drug bioavailability and patient compliance, particularly for those with difficulty swallowing tablets (contribution 10).

Lastly, the use of engineered extracellular vehicles (EVs) for stroke therapy is explored, highlighting their unique advantages such as the ability to cross the blood–brain barrier, target specific cells, and maintain stability in circulation (contribution 11). This review provides a forward-looking perspective on the challenges and future prospects of EVs in clinical settings, especially for treating neurological disorders. Together, these papers underscore the tremendous progress being made in developing sophisticated drug delivery systems that are not only more effective but also precisely tailored to the specific needs of various therapeutic areas, paving the way for more personalized and targeted medical treatments in the future.

## 3. Conclusions/Future Directions

The advances in polymeric delivery vehicles, as evidenced by recent studies, underscore the transformative potential of these systems in modern medicine. From the development of long-acting injectable formulations to the exploration of 3D-printed scaffolds and smart hydrogels, researchers are continually pushing the boundaries of what is possible in controlled and sustained drug release. As the field progresses, the integration of new materials, technologies, and design strategies will likely lead to even more innovative solutions, addressing unmet medical needs and improving patient outcomes. The studies discussed here provide a glimpse into the future of drug delivery, where precision, efficiency, and patient-centered design will be at the forefront of pharmaceutical development.
